# A Case Report of Geyser Sign on Magnetic Resonance Imaging (MRI) in a 65-Year-Old Female Patient

**DOI:** 10.7759/cureus.23751

**Published:** 2022-04-02

**Authors:** Ameer A Sayed, Mohammed Alariefy, Mohammed Aldawsari, Ahmed Nosair Aljedani, Hatem H Alharbi

**Affiliations:** 1 Department of Orthopaedics Surgery, King Fahad Armed Forces Hospital, Jeddah, SAU; 2 Department of Orthopaedics Surgery, King Fahad General Hospital, Jeddah, SAU; 3 Department of Orthopaedics Surgery, King Saud Bin Abdulaziz University for Health Sciences College of Medicine, Jeddah, SAU

**Keywords:** acromioclavicular joint, rotator cuff pathology, excision, surgery, geyser sign

## Abstract

The geyser sign is the flow of arthrographic contrast or joint fluid from the glenohumeral joint across the acromioclavicular joint (AC) and occurs when fluid erupts from the superior aspect of the AC joint during arthrography. The cyst’s pathogenesis is linked to a rotator cuff tear and an increase in the amount of fluid in the cyst. This fluid escapes through a one-way valve created by a defect in the AC joint capsule. The cysts, which are typically painless and rest over the AC joint, cause discomfort. We present a case of a 65-year-old female with a left shoulder mass. The patient presented to the outpatient department with a history of left shoulder mass for six months. She reported an increase in size with no constitutional symptoms. She was treated with surgical excision. Although rotator cuff tears and AC joint deterioration are rather common in medical practice, cystic swelling over the AC joint is a relatively uncommon symptom. Less than 50 cases have been reported to date. AC joint cyst is a mechanical consequence of a progressive and severe rotator cuff tear that can be misinterpreted as a tumor in older people. Imaging, particularly magnetic resonance imaging (MRI), should be used to rule out malignancy and make a precise diagnosis, including recognizing the “Geyser sign” if it is present.

## Introduction

The acromioclavicular (AC) joint cyst is an uncommon condition that arises as a result of a rotator cuff injury that persists. The cyst is made up of thick viscous fluid and is enclosed by a fibrous capsule. During arthrography, fluid emerges from the superior part of the AC joint, causing the geyser sign. A rotator cuff tear and an increase in the quantity of fluid in the rip are associated to the cyst’s etiology. A defect in the AC joint capsule creates a one-way valve, which allows the fluid to escape. Cysts, which are usually painless and above the AC joint, can be irritating [1]. Various diagnostic modalities, such as computed tomography (CT) scan, magnetic resonance imaging (MRI), arthrography, ultrasound, and others, have been used to diagnose AC joint cysts and rotator cuff injury with geyser sign. MRI is the first line of choice in the identification of AC joint cysts and rotator cuff injuries with geyser sign [2].

Type 1 and type 2 AC cysts are the most common. Degenerative AC joint alterations caused by trauma, infection, metabolic disorders, or overuse can lead to the formation of type 1 cysts. These cysts are only seen in the joint and aren’t linked to any rotator cuff issues. The synovium becomes inflamed as a result of long-term degenerative changes in the joint, resulting in their development. Some researchers suggest that the AC joint’s damaged capsule works as a valve, allowing the fluid to flow one-way from the subacromial bursa to the cyst [3,4].

Although rotator cuff tears and AC joint deterioration are rather common in medical practice, cystic swelling over the AC joint is a relatively uncommon symptom. Less than 50 cases have been documented in the literature to date. The majority of these cases were caused by a chronic rotator cuff injury, which resulted in glenohumeral joint fluid tracking into the subcutaneous planes via a connecting AC joint (type 2 cysts). The geyser sign was given to this leakage because it seemed to erupt as a geyser on a typical arthrogram. There have been fewer cases of type 1 cysts, which form as a result of AC joint degeneration with an intact rotator cuff. During a conventional arthrogram, the geyser sign was first described by Craig in 1984 as leakage of contrast from the glenohumeral joint into the subdeltoid bursa. However, as the use of MRI and musculoskeletal ultrasonography in the diagnosis of shoulder disorders has expanded, this symptom has become more commonly associated with chronic rotator cuff injuries [2,5,6]. We report a case of a female with a left shoulder mass with no previous history of tear or trauma.

## Case presentation

**Figure 1:**
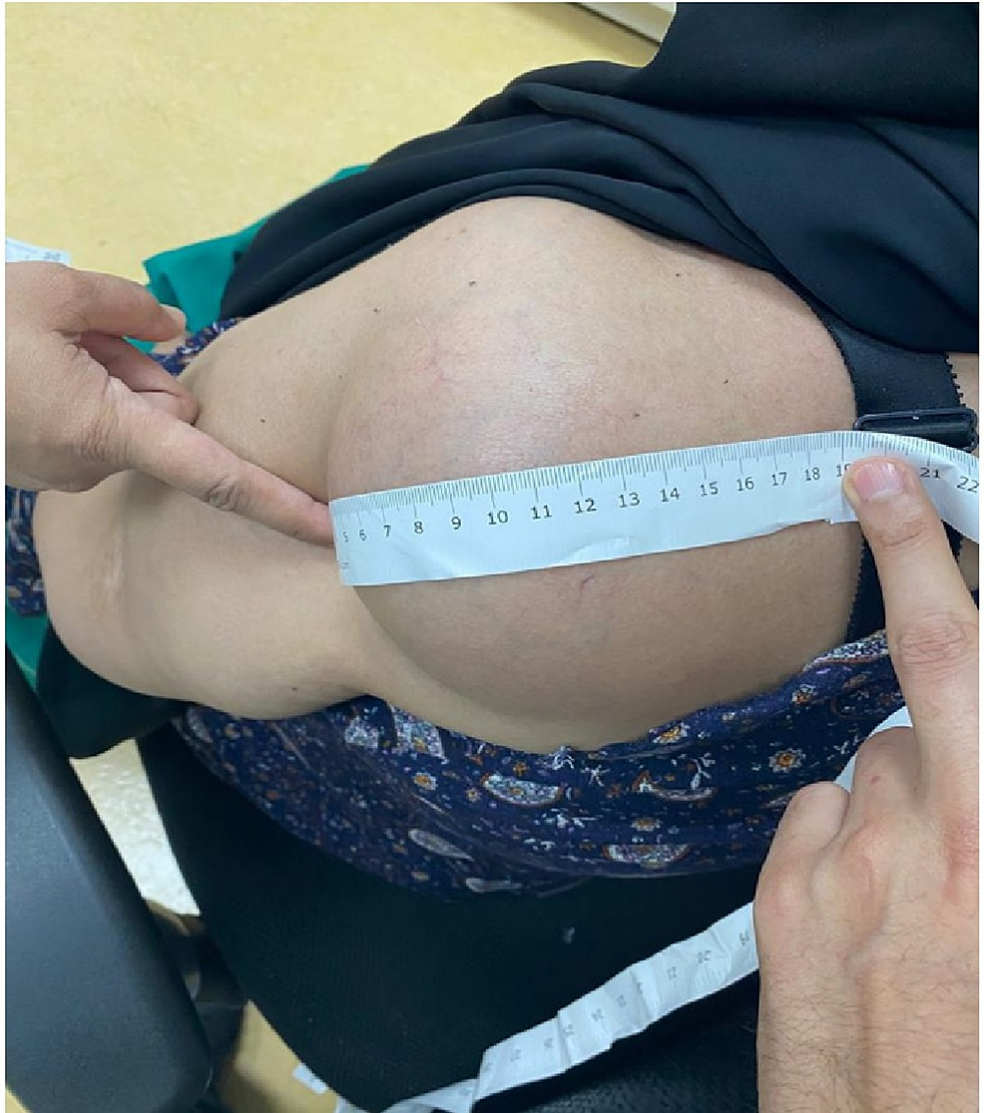
Mass of the left shoulder

A 65-year-old female patient not known to have any medical illness presented to the outpatient department with a history of left shoulder mass for six months. The mass was increasing in size. It was not associated with fever, weight loss, or night sweats. The patient denied any history of trauma to that region. On examination, there were no skin changes, no wounds, or erythema. She was found to have a soft, non-tender mass on the posterior aspect of the shoulder region. The swelling was mobile and not fixed to the underlying structure on examination (Figure 1).

**Figure 2:**
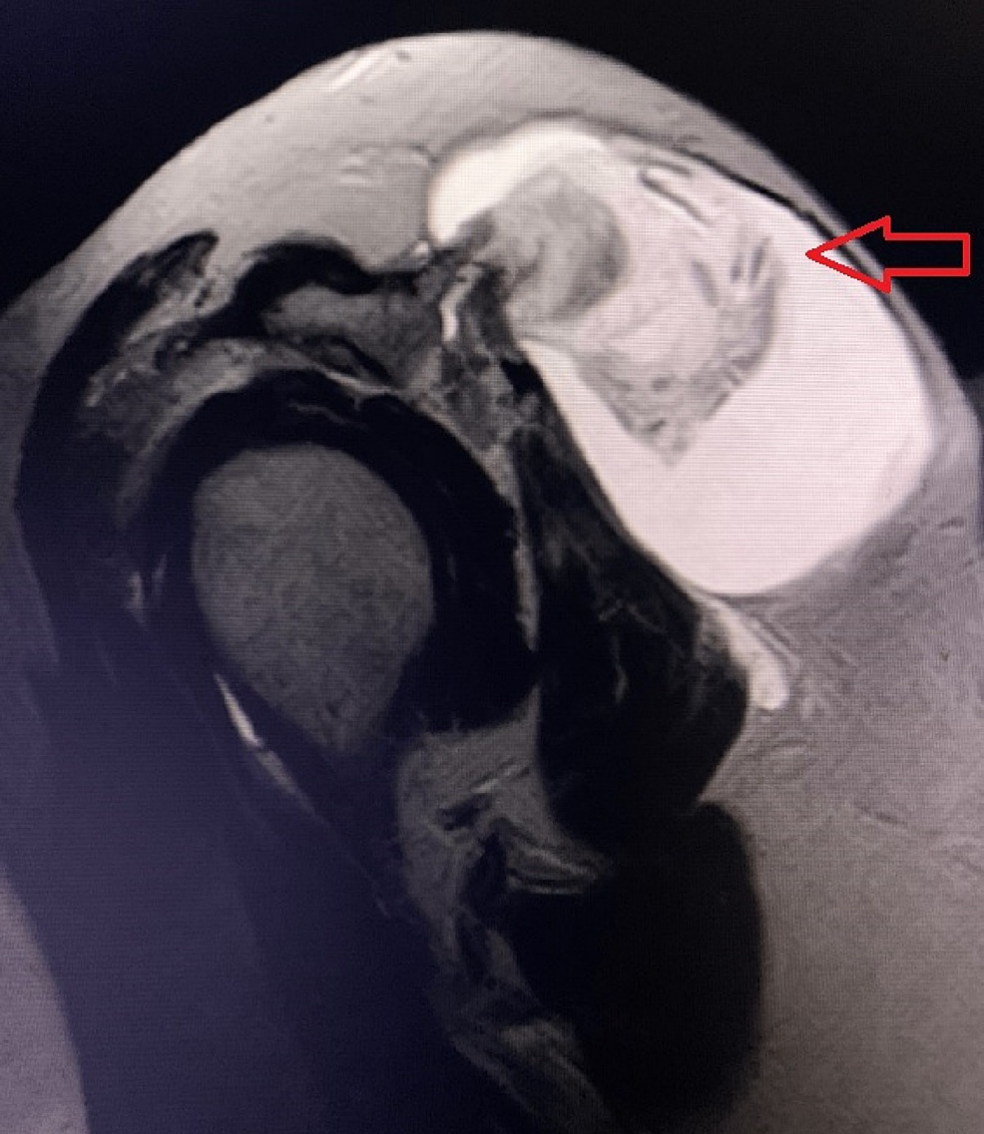
Magnetic resonance image of the left shoulder showing a cyst communicating with the glenohumeral joint fluid via a defect of the acromioclavicular joint (red arrow)

There was no hotness or signs of inflammation. There was no neurological deficit on examination and distal pulses were intact. Active and passive motions of the left shoulder revealed a restricted range of motion while Job’s and Hawkins’s tests were positive. The patient then underwent full laboratory and radiological investigations. The blood tests for the patient revealed a normal result except for the increase in C-reactive protein of 84.2 mg/L and erythrocytes sedimentation rate of 91 mm/h. Plain radiography revealed soft-tissue edema and elevation of the humeral head, as well as constriction of the glenohumeral joint space and decrease of the subacromial space. The patient also underwent a plain MRI of the left shoulder and showed AC joint arthritis with massive ganglion and partial supraspinatus tear (Figure 2).

Aspiration from the swelling revealed no evidence of infection or malignancy. The patient was then scheduled for surgical excision. The patient underwent an uneventful surgical excision. Samples were taken for histopathology and the results were consistent with bursitis and negative for malignancy and microorganisms. The cytology for the samples showed only inflammatory cells and negative for atypical or malignant cells. The impression of supraspinatus tear complicated by the formation of a cyst in the AC joint, the diagnosis of a geyser sign was made.

## Discussion

AC joint cysts are a rare consequence of severe AC joint arthritis or a persistent rotator cuff injury. They are classified as type-1 or type-2 cysts depending on the etiology. Type 1 cysts appear in advanced AC joint arthritis with synovial inflammation, are restricted to the joint, and are not linked to rotator cuff rupture [5]. The rotator cuff shape, on the other hand, governs the pathophysiology of a type 2 cyst. In rotator cuff arthropathy, a full rotator cuff rupture, particularly a supraspinatus tear, induces superior migration of the humeral head, causing irritation and degeneration of the AC joint capsule. Synovial fluid seeps into the AC joint capsule due to increased synovial fluid production and the creation of a check valve, leading to the formation of a ganglion cyst. In a shoulder arthrogram, Craig classified the pouring of fluid from the AC joint capsule as a geyser sign [6]. As a result, the fluid gathers beneath the skin and forms a cyst. The treating physician must be concerned about neoplasm at all times, which is an important reminder [7]. A patient with a significant AC joint cyst may have been evaluated for a neoplastic condition before presenting to an orthopedic surgeon; nonetheless, the orthopedist may be the first clinician to assess these patients. Current treatment recommendations for AC joint cysts recommend surgical surgery due to the danger of recurrence linked with aspiration [8,9]. To treat a solitary AC joint cyst with AC joint arthritis and an uninjured rotator cuff, Kontakis et al. conducted distal clavicular excision and subacromial bursectomy [3]. According to Skedros et al., an allograft patch was used to seal the surfaces of the resected bones and remaining AC joint ligaments, and the anterior deltoid was advanced to augment the excision site [10].

Conservative therapy is never indicated due to the presence of the check valve, which will continue to release the fluid through it. Our patient underwent surgical excision successfully. However, in literature, conservative care can include observation and needle aspiration with or without rotator cuff restoration. Meanwhile, surgical excision and glenohumeral joint hemiarthroplasty are more invasive treatment methods that have been shown to be effective in rapidly progressive cases [2]. When the mass is asymptomatic and the patient is unconcerned about the pain or appearance of the mass, observation may be appropriate. Repeated aspirations of these masses are generally not recommended, as they frequently reoccur, and repeated attempts at aspiration may result in the creation of a draining fistula, necessitating surgical intervention. Surgical excision has generally produced good results in terms of reduction of cyst recurrence. Although there have been quite a few reports of the AC joint cyst recurring following surgical excision, the majority of patients have reported positive outcomes. The quality and extent of surgical excision of the mass, the degree of resection of the AC joint or lateral border of the clavicle, the type of surgical closure performed, as well as patient-related factors that may be independent of the quality of surgery performed, may all contribute to this disparity [2].

Similarly, in 2015, Tucas and Kroon reported a case of an 87-year-old woman with a past medical history of osteoarthritis, rheumatoid arthritis, and left shoulder pain who presented with a four-week history of left shoulder swelling that has been spontaneous, painless, and growing. An MRI revealed a total thickness tear of the supraspinatus tendon, as well as retraction of the supraspinatus muscle. A soft-tissue tumor was discovered in the subcutaneous region cranial to the clavicle, passing between the rotator cuff tear and the enlarged AC joint showing the geyser sign. As a result of the disorder, muscle atrophy was observed [11]. Patel also reported a case of a 70-year-old woman in 2014 with geyser phenomenon who presented with swelling atop the AC joint, as well as pain and restricted shoulder movements for the last 6 months. Visible swelling above the AC joint, non-visualization of the long head biceps tendon, and a thin and aberrant echo pattern of the subscapularis tendon were all found during the investigation.

The supraspinatus and infraspinatus muscles bellies revealed considerable volume loss and fatty infiltration, with an acromiohumeral gap of 3 mm (normal: >6 mm) [12]. Maziak et al. in 2018 reported a case of a 77-year-old man who presented to the shoulder department with a soft-tissue tumor on his right AC joint. Prior puncture aspiration attempts had resulted in serous fluid retention, which had recurred after each drainage attempt. On conventional radiography and MRI of the affected shoulder joint, a progressed cuff-tear arthropathy with an irreversible supraspinatus tendon tear, static superior migration of the humeral head, opening of the AC joint capsule, and a superior joint fluid “eruption” and accumulation known as the “geyser sign” was revealed. An arthroscopic rotator cuff debridement and open cyst excision were performed due to the patient’s well-compensated cuff-tear arthropathy [13]. The AC joint cysts are benign processes that have varying pathophysiology depending on whether they are type 1 or type 2. On MRI, the presence of the geyser phenomenon in a type-2 cyst can be seen clearly without the necessity for a traditional shoulder arthrogram, distinguishing them from other shoulder diseases may aid in achieving final surgical therapy [14].

## Conclusions

AC joint cyst is a mechanical consequence of a progressive and severe rotator cuff tear that can be misinterpreted as a tumor in older people. Imaging, particularly MRI, should be used to rule out malignancy and make a precise diagnosis, including recognizing the geyser sign if it is present. We presented a case of a massive AC joint cyst in a 65-year-old woman with an advanced rotator cuff tear, abnormal shoulder function, and AC joint degeneration who was treated with surgical excision.
